# Radiological Alignment Trajectories and Late Functional Outcomes After Three-Level ACDF: A Single-Center Cohort Study

**DOI:** 10.3390/jcm15124739

**Published:** 2026-06-18

**Authors:** Merdan Orunoglu, Ukbe Sirayder, Oguzhan Yilmaz, Murat Baloglu

**Affiliations:** 1 Kayseri State Hospital Neurosurgery Clinic, Kocasinan 38010, Kayseri, Türkiye; 2 Department of Physiotherapy and Rehabilitation, Faculty of Health Sciences, Nuh Naci Yazgan University, Kocasinan 38170, Kayseri, Türkiye; usirayder@nny.edu.tr; 3 Department of Therapy and Rehabilitation, Vocational School of Higher Education, Nuh Naci Yazgan University, Kocasinan 38170, Kayseri, Türkiye; oguzhanylmz68@gmail.com; 4 Eskisehir City Hospital Neurosurgery Clinic, Odunpazari 26080, Eskisehir, Türkiye; mbalogluog@gmail.com

**Keywords:** three-level ACDF, cervical alignment, adjacent segment degeneration, neck disability index, quality of life

## Abstract

**Background**: Three-level anterior cervical discectomy and fusion (ACDF) is widely used for multilevel cervical degenerative disc disease; however, the relationship between postoperative alignment trajectories, adjacent segment degeneration (ASD), and late patient-reported outcomes remains incompletely defined. This study evaluated plane-specific radiological alignment changes, MRI-based ASD, and late functional outcomes in a homogeneous three-level ACDF cohort. **Methods**: This single-center observational cohort included 29 patients who underwent three-level ACDF between January 2018 and December 2023 and had complete radiographic follow-up. Radiological data were collected retrospectively from institutional records and imaging archives. Cervical sagittal and coronal alignment were assessed using Cobb angles on radiographs obtained preoperatively and at 6 months, 1 year, and 2 years postoperatively. ASD was evaluated at the superior adjacent segment on 2-year MRI. Late patient-reported clinical outcomes were assessed at a mean follow-up of 42.6 ± 6.8 months using the Visual Analog Scale (VAS), Neck Disability Index (NDI), and Nottingham Health Profile (NHP). **Results**: Sagittal Cobb angle changed significantly over time (χ^2^(3) = 12.60, *p* = 0.006; Kendall’s W = 0.145), whereas coronal Cobb angle showed a statistically significant reduction over time, although the absolute magnitude of change was small (χ^2^(3) = 28.74, *p* < 0.001; Kendall’s W = 0.330). Lower sagittal Cobb angle correlated with worse NDI (r = −0.46, *p* = 0.004), and greater coronal Cobb angle correlated with worse physical activity scores (r = 0.52, *p* = 0.006). Higher Pfirrmann grade correlated with worse NDI (r = 0.49, *p* = 0.004) and pain scores (r = 0.44, *p* = 0.021). In exploratory regression analysis, sagittal Cobb angle and Pfirrmann grade were retained in the model for NDI, but these findings should be interpreted as hypothesis-generating. **Conclusions**: After three-level ACDF, sagittal and coronal alignment followed different postoperative trajectories. Lower sagittal alignment and greater adjacent disc degeneration were associated with worse late neck-related disability. However, given the modest sample size and exploratory nature of the regression analysis, these findings should be interpreted as hypothesis-generating. Larger prospective studies are needed to confirm whether sagittal alignment and MRI-based adjacent segment degeneration independently contribute to late functional outcomes.

## 1. Introduction

Cervical degenerative disc disease, including cervical disc herniation and degenerative disc changes, is a common clinical condition that may cause radiculopathy or myelopathy affecting the neck and upper extremities, particularly with advancing age. In patients who do not respond to conservative treatment and meet surgical indications, anterior cervical discectomy and fusion (ACDF) is among the most frequently performed procedures. The principal goals of ACDF include decompression of neural structures via removal of the degenerated disc, restoration of segmental stability, and relief of clinical symptoms [1,2].

Beyond local decompression and stabilization, ACDF may influence global cervical biomechanics. Cervical sagittal alignment—particularly C2–C7 lordosis—and cervical coronal alignment are considered important for head posture, maintenance of horizontal gaze, overall spinal balance, and load distribution [3,4]. Preservation or restoration of cervical lordosis has therefore been evaluated not only as a radiographic target but also as a parameter potentially related to patient-reported outcomes and health-related quality of life [3,5].

While short-to-mid-term radiological alignment changes after single-level ACDF have been reported with relative consistency, multilevel ACDF—particularly three-level ACDF—introduces more complex biomechanical conditions. As the number of fused segments increases, alignment changes may vary across patients, and the clinical relevance of these changes remains debated [3,5]. In addition, although adjacent segment degeneration (ASD) has been described in terms of radiological progression and risk factors, its relationship with alignment parameters and long-term patient-reported outcomes after three-level ACDF remains incompletely characterized [4,5].

The novelty of the present study lies in its integrated assessment of plane-specific cervical alignment trajectories, MRI-based adjacent segment degeneration, and late disability-related patient-reported outcomes in a homogeneous three-level ACDF cohort. The present study aimed to (i) evaluate changes in cervical sagittal and coronal Cobb angles following three-level ACDF using serial radiographs up to 2 years, (ii) characterize radiological adjacent segment degeneration, and (iii) analyze associations between radiological findings at 2 years and late clinical outcomes and health-related quality of life assessed at final follow-up.

## 2. Materials and Methods

### 2.1. Study Design

This study was designed as a single-center observational cohort with retrospective radiological data collection and late patient-reported clinical outcome assessment. The study evaluated mid-term radiological alignment changes and adjacent segment degeneration after three-level anterior cervical discectomy and fusion (ACDF) and their association with late clinical outcomes.

### 2.2. Setting

Patients were identified from the institutional surgical database and medical records of a tertiary spine surgery practice. All eligible cases who underwent three-level ACDF for cervical disc herniation between January 2018 and December 2023 were screened. Radiological follow-up was conducted at predefined postoperative intervals, and late clinical outcomes were collected at the final follow-up visit.

Radiological data were obtained retrospectively from the institutional imaging archive at predefined time points: preoperatively and postoperatively at 6 months, 1 year, and 2 years. Late patient-reported outcomes were collected at final follow-up, either during outpatient control visits or through direct patient contact, at a mean of 42.6 ± 6.8 months after surgery.

### 2.3. Participants

Patients were eligible if they underwent three-level ACDF for symptomatic multilevel cervical disc herniation/degenerative disc disease with radiological neural compression, persistent symptoms despite conservative treatment, and radiographs available at the required follow-up time points (preoperative, postoperative 6 months, 1 year, and 2 years). Patients with primary cervical instability, traumatic pathology, infection, malignancy, prior cervical spine surgery, history of radiotherapy, deformity-correction indication, or insufficient clinical/imaging documentation for outcome assessment were excluded.

### 2.4. Study Size

All consecutive eligible patients within the study period were included. A total of 29 patients met the inclusion criteria and had complete radiographic follow-up. No a priori sample size calculation was performed because the study included all eligible patients within the defined study period; therefore, the cohort represents the total available eligible sample.

### 2.5. Surgical Procedure

All surgical procedures were performed in the operating room under general anesthesia with the patient in the supine position. The operative levels were confirmed preoperatively using fluoroscopic guidance. A standard right-sided transverse cervical incision was used in all patients. Following anterior cervical discectomy and neural decompression at the affected three levels, the endplates were prepared while preserving the bony endplate as much as possible. Interbody fusion was performed using bladed PEEK cages with titanium anchoring components. Local autologous graft material obtained from endplate preparation and osteophyte removal was placed within the cages. No anterior cervical plate fixation was used in any patient.

Postoperative fusion status was assessed during follow-up using plain radiographs and computed tomography when available. Fusion was defined radiologically by the presence of bridging bone and/or the absence of radiological signs of instability, with segmental motion of less than 2 mm on follow-up radiographic assessment.

### 2.6. Outcome Measurements

#### 2.6.1. Demographic and Physical Characteristics

Participants’ age, sex, height, body weight, and type of surgical procedure were recorded. Body mass index (BMI) was calculated by dividing weight in kilograms by height in meters squared.

#### 2.6.2. Quality of Life

Health-related quality of life was evaluated using the NHP, a validated tool designed to assess perceived health status. The Turkish version, which has been culturally adapted and psychometrically validated, was used in this study [6]. The questionnaire comprises subdomains including pain, emotional reactions, social isolation, sleep, energy level, and physical activity. Higher scores in each domain indicate greater impairment in that specific aspect of health.

#### 2.6.3. Functional Disability

Neck-specific functional disability was assessed using the Neck Disability Index (NDI), a widely used and validated questionnaire designed to evaluate functional limitations related to cervical spine disorders. The NDI consists of 10 items assessing pain intensity, personal care, lifting, reading, headaches, concentration, work, driving, sleeping, and recreation. Each item is scored on a 6-point scale ranging from 0 (no disability) to 5 (maximum disability), yielding a total score between 0 and 50. Total scores were converted to percentages, with higher scores indicating greater neck-related disability. The validated Turkish version of the NDI was used in this study [7].

#### 2.6.4. Pain Intensity

Neck pain intensity was assessed using the Visual Analog Scale (VAS), ranging from 0 to 10, where 0 indicated no pain and 10 indicated the worst imaginable pain [8].

#### 2.6.5. Radiological Alignment Measurements

**Figure 1:**
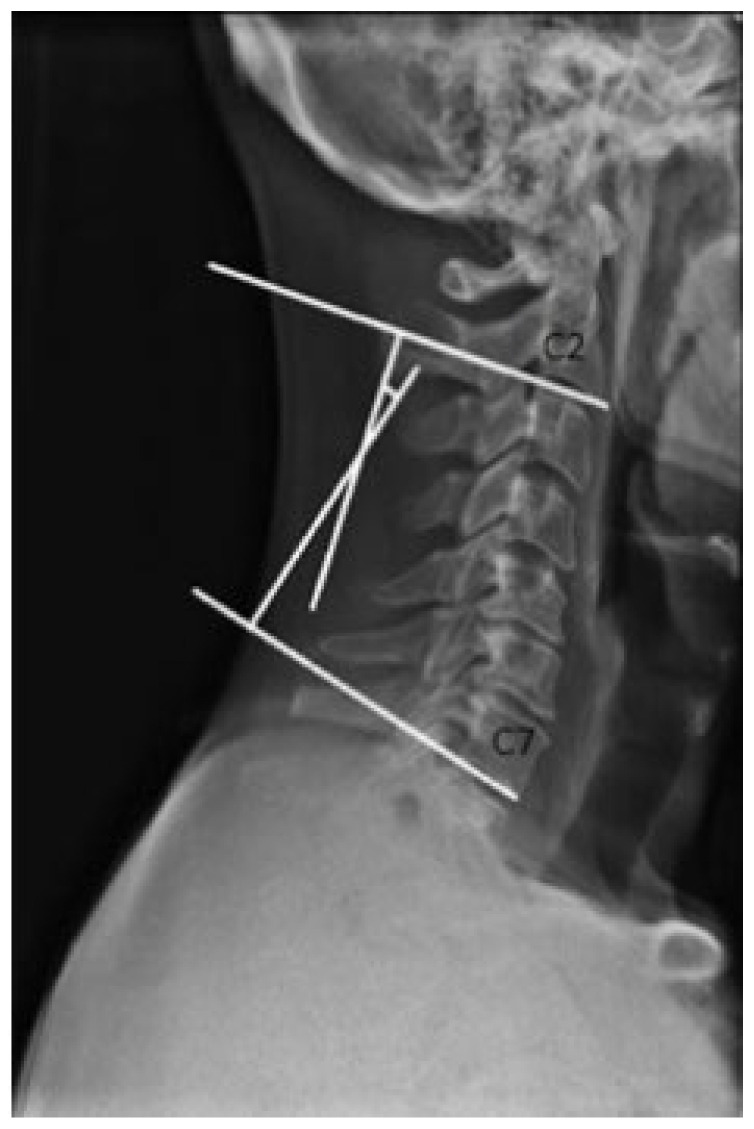
Measurement of cervical sagittal alignment using the C2–C7 Cobb angle.

Cervical sagittal alignment was assessed using the C2–C7 Cobb angle measured on lateral cervical radiographs. The angle was defined as the angle between lines drawn along the inferior endplates of the C2 and C7 vertebral bodies. Coronal cervical alignment was measured on anteroposterior radiographs using the coronal Cobb angle between the superior endplates of the uppermost and lowermost vertebrae within the measured segment [9]. Measurements were performed at four predefined time points: preoperatively and postoperatively at 6 months, 1 year, and 2 years. All radiographic measurements were performed using digital measurement tools within the institutional PACS system (AKGUN PACS, version 5.0.0.25, AKGUN Software, Ankara, Turkey). An illustration of the sagittal Cobb angle measurement method is shown in Figure 1.

#### 2.6.6. Radiological Assessment of Adjacent Segment Disease (ASD)

ASD was evaluated at the intervertebral level immediately proximal to the fused segment. Radiological assessment included three parameters:

- ***Disc Degeneration:*** Assessed using the Pfirrmann grading system based on MRI signal intensity, contour integrity, and disc height of the adjacent superior disc [10,11].
- ***Disc Height Loss:*** Measured on sagittal T2-weighted MRI sequences by calculating the intervertebral height between adjacent vertebral bodies. A reduction greater than 25% was considered indicative of degeneration [12].
- ***Facet Joint Degeneration:*** Evaluated using axial CT or T2-weighted MRI images and classified according to the Weishaupt grading system [13].

All imaging measurements were performed by a single experienced radiologist who was blinded to clinical outcomes. Digital measurement tools integrated into the PACS system were used for all evaluations. Because MRI data beyond 2 years were not available in the institutional records, ASD parameters were evaluated using the 2-year postoperative MRI as the latest radiological reference. Because late patient-reported outcomes were collected at a mean of 42.6 ± 6.8 months after surgery, the 2-year MRI assessment and final clinical outcome assessment were not temporally synchronized. Therefore, ASD parameters were interpreted as radiological findings at the 2-year postoperative time point rather than as imaging findings obtained concurrently with the final VAS, NDI, and NHP assessments.

### 2.7. Statistical Analysis

Statistical analyses were performed using IBM SPSS Statistics (Version 27.0; IBM Corp., Armonk, NY, USA). Continuous variables were expressed as mean ± standard deviation (SD) or median (interquartile range, IQR), as appropriate. Normality was assessed using the Shapiro–Wilk test.

Longitudinal changes in sagittal and coronal Cobb angles were analyzed using the Friedman test. When significant, post hoc pairwise comparisons were performed using the Wilcoxon signed-rank test with Bonferroni correction (adjusted significance level *p* < 0.008 for six comparisons). Effect sizes were calculated using Kendall’s W for Friedman tests and r = Z/√N for pairwise comparisons.

Associations between radiological parameters at 2 years and clinical outcomes at final follow-up were evaluated using Spearman rank correlation coefficients. To account for multiple testing (seven correlations), Bonferroni correction was applied (adjusted significance level *p* < 0.007).

Given the modest sample size, the multivariable linear regression analysis was conducted as an exploratory and hypothesis-generating analysis rather than as a confirmatory predictive model. Variables showing an association with NDI at *p* < 0.10 in univariate analyses were entered into the model to explore whether radiological parameters provided complementary information regarding late neck-related disability. Model fit was assessed using R^2^ and adjusted R^2^, and multicollinearity was evaluated using variance inflation factor (VIF). The regression findings were interpreted cautiously due to the limited number of observations and the potential risk of model instability and overfitting. All tests were two-tailed, and *p* < 0.05 was considered statistically significant unless otherwise specified.

## 3. Results

**Figure 2:**
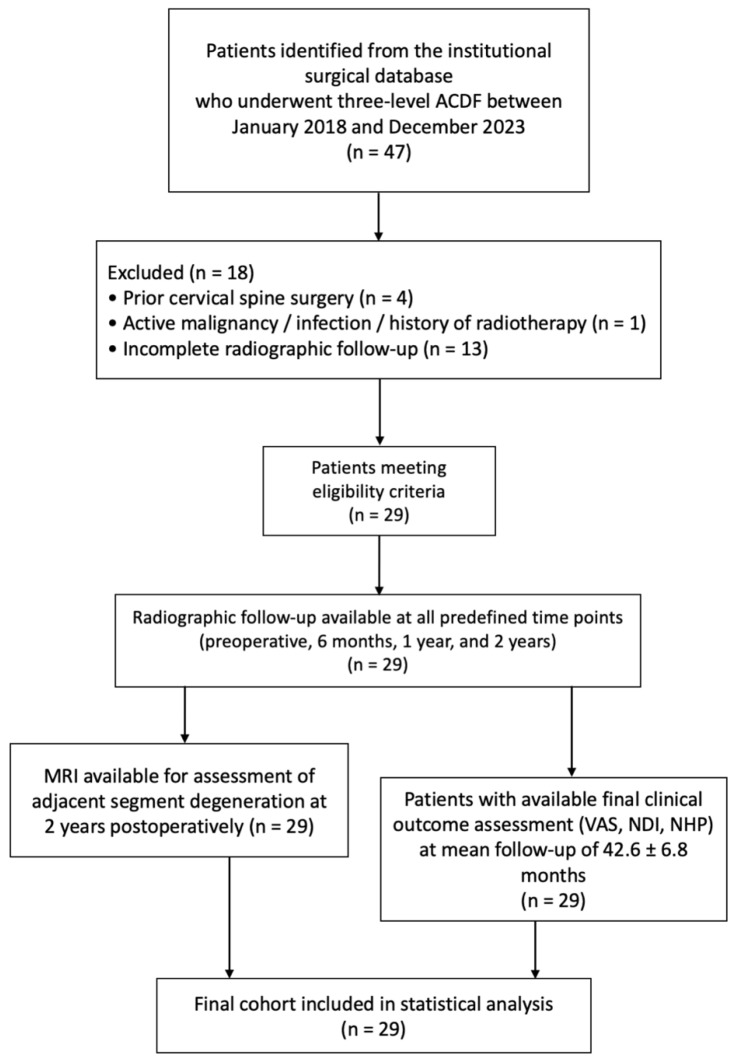
Flow diagram of patient identification, eligibility screening, radiographic follow-up, and final inclusion in the statistical analysis for patients undergoing three-level ACDF.

A total of 47 patients who underwent three-level anterior cervical discectomy and fusion (ACDF) between January 2018 and December 2023 were initially identified from the institutional surgical database. Of these, 18 patients were excluded: 4 due to prior cervical spine surgery, 1 due to active malignancy/infection or a history of radiotherapy, and 13 due to incomplete radiographic follow-up. The remaining 29 patients met the eligibility criteria and had complete radiographic follow-up at all predefined time points (preoperative, 6 months, 1 year, and 2 years). MRI examinations at 2 years postoperatively were available for assessment of adjacent segment degeneration in all included patients. Final clinical outcome assessment, including VAS, NDI, and NHP, was available at a mean follow-up of 42.6 ± 6.8 months for all patients. The final cohort included in the statistical analysis consisted of 29 patients (Figure 2). The operated levels were C4–C6 in 23 patients (79.3%), C3–C5 in 4 patients (13.8%), and C5–C7 in 2 patients (6.9%). Review of the available clinical records between the 2-year MRI assessment and the final patient-reported outcome assessment showed no documented revision cervical surgery or additional cervical spine intervention during this interval. However, minor symptom fluctuations, conservative treatment exposure, or radiological progression not captured by repeat MRI could not be completely excluded.

At a mean follow-up of 42.6 ± 6.8 months, clinical outcomes at final follow-up are summarized in Table 1. The mean VAS score for neck pain was 2.8 ± 1.6, and the mean Neck Disability Index (NDI) score was 23.24 ± 6.35%. Among the Nottingham Health Profile (NHP) domains, the highest mean score was observed in the sleep domain (22.37 ± 27.79), while physical activity (2.24 ± 5.35) and social isolation (2.75 ± 6.13) demonstrated lower values.

**Figure 3:**
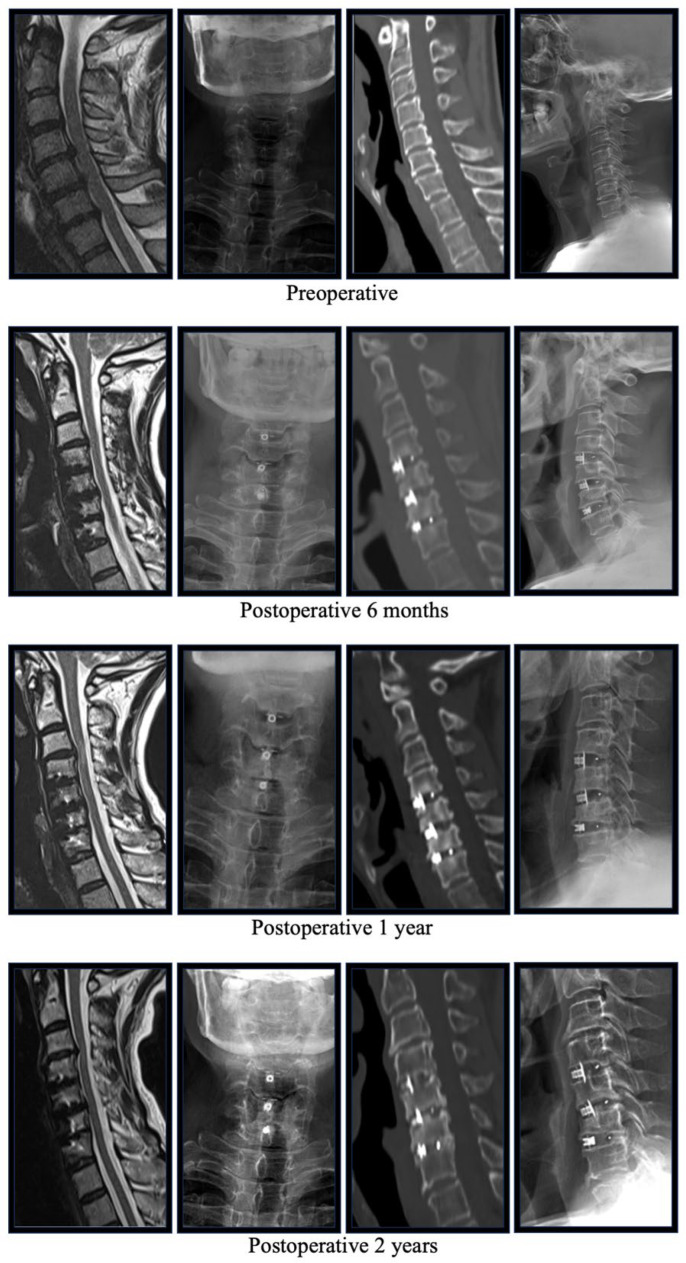
Representative radiological follow-up of a patient undergoing three-level anterior cervical discectomy and fusion (ACDF).

Longitudinal changes in sagittal and coronal Cobb angles are shown in Table 2. A representative example of longitudinal radiological changes following three-level ACDF is shown in Figure 3. The Friedman test demonstrated a significant overall time effect for sagittal Cobb angle (χ^2^(3) = 12.60, *p* = 0.006), with a Kendall’s W of 0.145. Post hoc pairwise comparisons using Wilcoxon signed-rank tests with Bonferroni correction (adjusted α = 0.008) showed a significant difference between 6 months and 2 years postoperatively (Z = −2.876, *p* = 0.004, r = 0.53). No other pairwise comparisons were statistically significant (Table 3).

For coronal Cobb angle, Friedman analysis revealed a significant time effect (χ^2^(3) = 28.74, *p* < 0.001), with a Kendall’s W of 0.330. Post hoc pairwise comparisons demonstrated significant differences between 2 years and preoperative measurements (Z = −3.687, *p* < 0.001, r = 0.69), 2 years and 6 months (Z = −3.709, *p* < 0.001, r = 0.69), and 2 years and 1 year (Z = −4.110, *p* < 0.001, r = 0.76).

Spearman correlation analyses between radiological parameters at 2 years and clinical outcomes at final follow-up are presented in Table 4. Sagittal Cobb angle was negatively correlated with NDI (r = −0.46, *p* = 0.004). Coronal Cobb angle was positively correlated with NHP physical activity (r = 0.52, *p* = 0.006). Pfirrmann grade was positively correlated with NDI (r = 0.49, *p* = 0.004) and NHP pain (r = 0.44, *p* = 0.021). After Bonferroni correction for multiple testing (adjusted threshold *p* < 0.007), the correlations between sagittal Cobb angle and NDI, coronal Cobb angle and NHP physical activity, and Pfirrmann grade and NDI remained statistically significant. Disc height loss and facet degeneration score were not significantly correlated with NDI.

An exploratory multiple linear regression analysis was performed to evaluate whether radiological parameters at 2 years showed complementary associations with NDI at final follow-up (Table 5). Variables showing an association with NDI at *p* < 0.10 in univariate analyses were entered into the model. The exploratory model was statistically significant (F = 6.89, *p* = 0.004), explaining 42% of the variance in NDI scores (R^2^ = 0.42; adjusted R^2^ = 0.37). Sagittal Cobb angle at 2 years (B = −0.58, β = −0.39, *p* = 0.025) and Pfirrmann grade (B = 3.12, β = 0.44, *p* = 0.009) were retained in the model. VIF values were below 2 for all variables.

## 4. Discussion

The present study evaluated mid-term radiological changes and late clinical outcomes after three-level ACDF in a single surgical cohort. The main findings were as follows: first, clinical outcomes at final follow-up suggested relatively low residual neck pain and mild-to-moderate disability; second, sagittal Cobb angle changed significantly over time, although with a small effect size and with the only significant pairwise difference occurring between 6 months and 2 years; third, coronal Cobb angle showed a more consistent and progressive reduction over follow-up, with a moderate effect size; fourth, greater adjacent segment degeneration, particularly higher Pfirrmann grade, was associated with worse disability and pain-related quality-of-life scores; and finally, in an exploratory multivariable regression model, lower sagittal Cobb angle and higher Pfirrmann grade were retained as variables associated with NDI at final follow-up. Taken together, these findings suggest that after three-level ACDF, radiological alignment and adjacent segment degeneration may provide complementary information regarding patient-reported outcomes; however, the regression findings should be interpreted as exploratory and hypothesis-generating rather than confirmatory.

With respect to overall clinical status, our cohort demonstrated relatively favorable late outcomes, with low mean VAS scores and NDI values in the mild-to-moderate range. This is generally consistent with recent multilevel ACDF literature reporting that meaningful symptom relief and functional improvement can still be achieved despite the greater biomechanical burden associated with longer fusion constructs. Recent comparative and long-term series have similarly shown that multilevel ACDF can provide satisfactory late patient-reported outcomes, although outcome variability appears to increase as the number of fused levels rises [14,15]. This pattern is important because it suggests that acceptable late disability and quality-of-life status may coexist with modest radiological deterioration over time.

Regarding sagittal alignment, our data showed a significant overall time effect for the sagittal Cobb angle, but the effect size was small and the only statistically robust pairwise difference was between 6 months and 2 years. This pattern does not support a durable progressive increase in lordosis over time; rather, it suggests a transient early postoperative improvement followed by partial loss during follow-up. Such a trajectory is broadly in line with more recent literature indicating that multilevel ACDF may initially restore or improve cervical lordosis, but that this gain is not always fully maintained, especially as the number of fused segments increases [16]. In a recent comparative study including one-, two-, and three-level ACDF procedures, postoperative radiological improvement was observed across groups, but the three-level cohort remained biomechanically more vulnerable than shorter constructs [17]. Likewise, newer work on multilevel ACDF has emphasized that alignment correction may occur together with evolving compensatory changes rather than with stable long-term maintenance of all sagittal parameters [5].

Our findings also fit with prior observations that three-level constructs may have a limited ability to preserve lordosis over time compared with shorter fusions. Earlier comparative studies reported that as fusion length increases, postoperative lordotic restoration becomes less stable and the risk of later flattening becomes more apparent. In that prospective cohort, although postoperative lordosis improved initially, patients undergoing three-level ACDF demonstrated a gradual decrease in C2–C7 lordosis during follow-up, declining from approximately 25.6° in the early postoperative period to about 20.3° at final follow-up (*p* = 0.001) [17]. More recent analyses have extended this concept by suggesting that multilevel ACDF should be viewed not simply as a local segmental correction procedure, but as an intervention that redistributes alignment demands across fused and unfused cervical segments within the global spinal alignment framework [18]. From a mechanistic standpoint, once three contiguous levels are fused, the fused segment may gain immediate structural alignment, but the remaining mobile segments may subsequently participate in compensation, which can make the initial postoperative lordosis less durable at later assessments. In addition, recent surgical outcome analyses have suggested that increasing fusion length in ACDF procedures may also be associated with longer operative times, greater intraoperative blood loss, and higher complication rates, including instrumentation-related problems and adjacent segment changes [15]. Similarly, a recent review of four-level ACDF reported that multilevel anterior cervical fusion can be clinically effective but remains associated with relevant complication and revision concerns, supporting the need for cautious interpretation of outcomes as fusion length increases [19]. These factors may further contribute to the complex postoperative biomechanical environment observed after multilevel fusion constructs.

In contrast, coronal Cobb angle showed a statistically significant reduction over time, with a moderate Kendall’s W and several significant pairwise comparisons. However, the absolute magnitude of coronal Cobb values was small, ranging from 1.99° preoperatively to 1.43° at 2 years. Therefore, although this change was statistically detectable in the present dataset, its clinical significance is uncertain. These sub-2° differences may fall within the expected range of technical and observer-related measurement variability for plain radiographic Cobb angle assessment and may also reflect regression to the mean rather than a true biomechanical correction. Accordingly, the coronal Cobb findings should be interpreted cautiously and should not be considered definitive evidence of clinically meaningful coronal realignment after three-level ACDF.

The association between radiological alignment and patient-reported outcomes in our study was selective rather than global. Lower sagittal Cobb angle at 2 years was associated with worse NDI and greater neck pain, while greater coronal Cobb angle was associated with worse NHP physical activity scores. These findings suggest that residual malalignment may be reflected more clearly in specific functional domains than in global quality-of-life summaries. This selective pattern is also compatible with prior literature indicating that cervical alignment parameters do not always correlate uniformly with all clinical endpoints after ACDF [20]. Rather, certain domains—particularly disability and activity-related function—may be more sensitive to residual or evolving alignment changes than others. From a biomechanical perspective, this is plausible because reduced lordosis or persistent coronal asymmetry may alter muscular demand, head posture, and load sharing across the cervical spine without necessarily translating into uniformly worse scores across every outcome scale.

An important exploratory observation of the current study is that adjacent segment degeneration, especially disc degeneration reflected by higher Pfirrmann grade, was associated with worse late disability and pain-related quality-of-life scores. This is one of the most important clinical messages of the study. Recent systematic evidence has highlighted postoperative cervical alignment, change in sagittal parameters, and adjacent-level biomechanics as relevant radiographic factors in the development of ASD after ACDF. However, relatively few studies have directly examined the relationship between radiographic adjacent segment changes and late patient-reported disability specifically in cohorts undergoing three-level ACDF [5,15]. Our results suggest that not all radiographic ASD markers carry equal clinical weight: Pfirrmann grade showed a meaningful association with NDI and NHP pain, whereas disc height loss and facet degeneration did not reach statistical significance. This may indicate that qualitative disc degeneration on MRI captures clinically relevant adjacent segment burden more sensitively than a single structural dimension such as height loss alone. However, because ASD was assessed at 2 years and clinical outcomes were obtained later, this association should be interpreted cautiously. The available data do not allow us to determine whether ASD progressed during the interval or whether interval clinical factors influenced the final disability and quality-of-life scores.

The relationship between alignment and ASD after three-level ACDF has remained controversial, and our data add nuance to that discussion. A prior three-level ACDF study specifically examining sagittal balance and ASD reported that cervical sagittal alignment was not clearly associated with ASD development in that setting. By contrast, the present study does not claim that malalignment leads to ASD, but rather shows that lower sagittal Cobb angle and greater adjacent disc degeneration coexist with worse late disability [4]. These are related but not identical observations. One concerns whether alignment predicts ASD occurrence; the other concerns whether alignment status and ASD severity are each associated with clinical status at later follow-up. Our data therefore support a more integrated interpretation in which radiological alignment and adjacent segment degeneration may represent parallel dimensions of postoperative biomechanical adaptation, both of which are clinically relevant even when one does not fully explain the other.

In the exploratory multivariable regression analysis, sagittal Cobb angle and Pfirrmann grade were both retained in the model for NDI at final follow-up. However, because the cohort included only 29 patients, this finding should not be interpreted as confirmatory evidence that these variables are independent predictors of late disability. Rather, the result suggests that sagittal alignment and adjacent disc degeneration may provide complementary information regarding patient-reported neck-related disability after three-level ACDF. This preliminary observation should be considered hypothesis-generating and requires validation in larger prospective cohorts with adequate statistical power.

The present findings also have practical implications for postoperative evaluation and rehabilitation-oriented follow-up. In routine clinical practice, postoperative success after ACDF is often summarized in terms of decompression, fusion, and immediate symptom relief. Our results suggest that in three-level constructs, longer-term follow-up may benefit from including both alignment surveillance and adjacent segment MRI-based assessment, particularly when disability remains higher than expected. From a rehabilitation perspective, the link between radiological parameters and disability/activity domains supports an approach in which persistent functional limitations are interpreted in conjunction with postoperative biomechanics rather than only with pain intensity. This is especially relevant in multilevel fusions, where the cervical spine’s capacity for motion redistribution is inherently reduced.

Several limitations should be acknowledged. First, the single-center observational design, retrospective radiological data collection, and exclusion of 13 patients with incomplete radiographic follow-up limit external generalizability and may have introduced selection bias; because detailed follow-up data were unavailable for excluded patients, formal comparison with the included cohort could not be performed. Second, the modest sample size limited the number of variables suitable for multivariable modeling and increased the risk of model instability; therefore, the regression findings should be considered exploratory. Third, preoperative VAS, NDI, and NHP scores were not available for all patients, so clinical improvement from baseline could not be quantified and the clinical results should be interpreted as late postoperative status rather than treatment-related change. Fourth, ASD was assessed using 2-year postoperative MRI, whereas patient-reported outcomes were collected later, creating temporal discordance that precludes direct concurrent or causal radiological–clinical interpretation. Fifth, radiological analysis was limited to Cobb-based alignment assessment and did not include broader sagittal balance or construct-specific parameters such as C2–C7 SVA, T1 slope, segmental lordosis, or detailed fusion grading. Finally, all radiological measurements were performed by a single radiologist without formal intra- or interobserver reliability testing, which limits reproducibility, particularly for semi-quantitative grading systems and small-magnitude coronal Cobb angle changes.

## 5. Conclusions

The present study suggests that after three-level ACDF, sagittal and coronal Cobb angles may show different postoperative patterns; however, the small absolute magnitude of coronal Cobb angle change limits its clinical interpretation. Greater adjacent segment disc degeneration on 2-year MRI and lower sagittal Cobb angle were associated with worse late neck-related disability. However, because MRI-based ASD assessment and patient-reported outcomes were not temporally synchronized, and because radiological measurements were performed by a single observer without formal reliability testing, these associations should be interpreted cautiously. Larger prospective studies with concurrent radiological and clinical follow-up and formal measurement reliability assessment are needed to clarify the relationship between postoperative alignment, adjacent segment degeneration, and late functional outcomes.

## Figures and Tables

**Table 1:** Clinical and Functional Outcomes at Final Follow-up.

Parameter	Value
Follow-up duration (months)	42.6 ± 6.8
VAS—Neck pain	2.8 ± 1.6
Neck Disability Index (NDI, %)	23.24 ± 6.35
NHP—Pain	5.18 ± 7.64
NHP—Physical Activity	2.24 ± 5.35
NHP—Energy Level	4.63 ± 11.97
NHP—Sleep	22.37 ± 27.79
NHP—Emotional Reactions	8.07 ± 11.61
NHP—Social Isolation	2.75 ± 6.13

Values are presented as mean ± standard deviation. Clinical and functional outcomes were assessed at the final follow-up visit conducted at a mean follow-up of 42.6 ± 6.8 months after surgery.

**Table 2:** Longitudinal Changes in Sagittal and Coronal Cobb Angles Following Three-Level ACDF (n = 29).

Time Point	Sagittal Cobb (°) Mean ± SD	Median (IQR)	Coronal Cobb (°) Mean ± SD	Median (IQR)
Preoperative	16.56 ± 3.81	16.24 (14.02–17.51)	1.99 ± 0.53	1.94 (1.66–2.34)
Postoperative 6 months	17.11 ± 4.60	17.49 (14.45–20.21)	1.77 ± 0.36	1.93 (1.46–2.08)
Postoperative 1 year	15.97 ± 3.69	15.87 (13.16–19.08)	1.70 ± 0.38	1.81 (1.35–1.94)
Postoperative 2 years	15.31 ± 4.04	14.48 (13.36–17.71)	1.43 ± 0.42	1.39 (1.19–1.80)

Friedman test: Sagittal χ^2^(3) = 12.60, *p* = 0.006, Kendall’s W = 0.145; Coronal χ^2^(3) = 28.74, *p* < 0.001, Kendall’s W = 0.330.

**Table 3:** Post hoc Pairwise Comparisons Following Friedman Test (Bonferroni corrected).

Comparison	Sagittal *p*_adj	Sagittal r	Coronal *p*_adj	Coronal r
Preop—6 m	0.469	0.13	0.066	0.34
Preop—1 y	0.880	0.03	0.009	0.49
Preop—2 y	0.110	0.30	<0.001 *	0.69
6 m—1 y	0.058	0.35	0.098	0.31
6 m—2 y	0.004 *	0.53	<0.001 *	0.69
1 y—2 y	0.094	0.31	<0.001 *	0.76

* Significant after Bonferroni correction (α = 0.008).

**Table 4:** Correlations Between Radiological Parameters at 2 Years and Clinical Outcomes at Final Follow-up.

Radiological Parameter (2 Years)	Clinical Parameter (3–4 Years)	r	*p*	Bonferroni-Corrected
Sagittal Cobb angle	NDI (%)	−0.46	0.004	Yes
Sagittal Cobb angle	VAS—Neck pain	−0.41	0.028	No
Coronal Cobb angle	NHP—Physical Activity	0.52	0.006	Yes
Pfirrmann grade (ASD)	NDI (%)	0.49	0.004	Yes
Pfirrmann grade (ASD)	NHP—Pain	0.44	0.021	No
Disc height loss (%)	NDI (%)	0.19	0.320	No
Facet degeneration score	NDI (%)	0.38	0.056	No

Spearman rank correlation coefficient was used. Bonferroni-adjusted significance threshold: *p* < 0.007.

**Table 5:** Multiple Linear Regression Analysis of Factors Associated with Neck Disability Index at Final Follow-up.

Variable	B	SE	β	95% CI	*p*
Sagittal Cobb (2 y)	−0.58	0.24	−0.39	−1.08 to −0.08	0.025
Pfirrmann Grade (2 y)	3.12	1.10	0.44	0.85 to 5.39	0.009

Model statistics: R^2^ = 0.42, Adjusted R^2^ = 0.37, F = 6.89, *p* = 0.004, VIF < 2 for all variables.

## Data Availability

The anonymized dataset supporting the findings of this study has been provided to the editorial office for internal evaluation in a redacted form due to patient confidentiality, institutional data protection requirements, and prevention of potential misuse. Additional information related to the dataset may be obtained from the corresponding author upon reasonable request and subject to institutional approval.
